# Tailoring Chronic Disease Interventions to Meet Specific Needs of Women: A Case Example of a Hypertension Program

**DOI:** 10.1089/whr.2024.0139

**Published:** 2025-03-07

**Authors:** Caroline A. Dancu, Julie Schexnayder, Hayden B. Bosworth, Allison Lewinski, Abigail Shapiro, Tiera Lanford, Courtney White Clark, Bevanne Bean-Mayberry, Leah L. Zullig, Jennifer M. Gierisch, Karen M. Goldstein

**Affiliations:** ^1^ San Francisco School of Nursing, University of California, San Francisco, California, USA.; ^2^ San Francisco Veterans Affairs Medical Center, San Francisco, California, USA.; ^3^ University of Alabama School of Nursing, Birmingham, Alabama, USA.; ^4^ Durham Center of Innovation to Accelerate Discovery and Practice Transformation, Durham Veterans Affairs Health Care System, Durham, North Carolina, USA.; ^5^ Department of Population Health Sciences, Duke University School of Medicine, Durham, North Carolina, USA.; ^6^ Duke University School of Nursing, Durham, North Carolina, USA.; ^7^ Department of Medicine, Duke University School of Medicine, Durham, North Carolina, USA.; ^8^ Center for the Study of Healthcare Innovation, Implementation & Policy (CSHIIP), VA Greater Los Angeles Healthcare System, Los Angeles, California, USA.; ^9^ Department of Medicine, UCLA David Geffen School of Medicine, Los Angeles, California, USA.

**Keywords:** cardiovascular risk, sex- and gender-tailoring, Veterans

## Abstract

**Background:**

Women have a unique risk profile for cardiovascular disease (CVD) due to underlying sociocultural and biological determinants. Current CVD prevention and treatment interventions, however, largely remain agnostic to the influences of an individual’s sex assigned at birth or gender identity. This study describes a process for tailoring existing evidence-based interventions to the biological and sociocultural determinants of health for women.

**Methods:**

This study adapted the Team-supported, Electronic Health Record (EHR)-leveraged, Active Management (TEAM) CVD preventative care intervention designed for telehealth-based remote hypertension (HTN) care in rural Veterans. Tailoring choices were informed by a 12-month process including a focused literature review, qualitative interviews with women’s health experts, and feedback from providers and women Veterans on existing intervention materials.

**Results:**

Literature review and qualitative interview findings informed the modification of patient- and provider-facing TEAM materials. Patient-facing material modifications included the addition of information relevant to sex-specific CVD risk factors, addressing gender-related barriers to CVD risk reduction, and including diverse visual representation and inclusive language. Provider-facing materials were modified through a new EHR template to comprehensively address sex-specific CVD risk factors. These changes resulted in individualized care plans to better address gaps in HTN management among women.

**Conclusion:**

Tailoring existing evidence-based interventions is an achievable and practical strategy to incorporate the sociocultural and biological determinants of CVD health specific to women. This approach could be used to adapt other programs and interventions designed to address health conditions that occur among both men and women but which are sensitive to important biological and sociocultural determinants. These findings highlight the broad discourse on sex- and gender-sensitive health care interventions and advocate for the integration of these interventions into routine clinical practice.

## Introduction

Cardiovascular disease (CVD) remains the leading cause of mortality among men and women in the United States.^1^ However, women face unique challenges across the continuum of CVD care, including lower risk awareness, underutilization of preventive strategies, delayed diagnoses, and disparities in treatment. CVD risk assessment, for example, is substantially less common among women than men,^2,3^ as in the administration of guideline-directed medical therapy (GDMT) for heart failure.^4^ In particular, CVD risk determinants due to underlying biological differences based on sex assigned at birth are often overlooked (*e.g.,* hormone shifts with menopause and pregnancy-related CVD diagnoses). Indeed, although many CVD-based interventions have been established,^5^ they often fail to address important factors related to an individual’s sex (*i.e.,* a biological construct based on anatomy, physiology, genetics, and hormones) and gender (*i.e.,* a sociocultural construct encompassing gender identity and expression).^6^ Failing to incorporate important sex-specific and gender-related health care considerations that are unique to women leads to inequitable opportunities for optimal, patient-centered care given the importance of both biology and sociocultural influences on CVD risk.^7,8^ Thus, tailoring CVD risk reduction interventions to the specific biological and sociocultural determinants of women is critical.

Women Veterans (WV) have unique cardiovascular risk profiles and warrant distinct CVD risk reduction efforts.^9^ WV represent the fastest-growing population of Veterans in the US, rising from 4% of the overall Veteran population in 2000 to a projected 18% by 2040.^10^ WV have higher rates of CVD^11^ and CVD-associated mortality than civilian women,^9^ as well as higher prevalence of certain CVD risk factors (*e.g.,* depression, obesity) compared with male Veterans (MV).^12^ WV are also more racially diverse than MV, highlighting structural racism as a potential additive CVD risk factor among WV of color.^13^ Inequitable CVD health care practices persist between women and men veterans. For example, WV who participated in recent military conflicts and those aged <55 years are less likely to receive GDMT after a diagnosis of coronary artery disease or heart failure than MV.^14^ Furthermore, WV experience a higher burden of sociocultural experiences, such as military sexual trauma and caregiving responsibilities which present additional CVD risk and challenges to CVD risk management.^15–17^ Moreover, some WV have attributed provider misperception of CVD risk as an additional barrier to preventative behavior engagement.^18^ Collectively, these data indicate the prevalence of implicit bias and gender-specific barriers to the receipt of high-quality CVD risk reduction for WV.

Despite the multiple contributors and potential challenges to optimizing CVD risk management for WV, opportunities for intervention exist. For example, WV attend more primary care visits than MV,^12^ suggesting the greater potential for opportunities to engage around comprehensive CVD assessment and prevention interventions. The Veterans Health Administration (VA) has made multiple investments around CVD risk reduction efforts for WV, including partnering with the American Heart Association’s (AHA) “Go Red for Women” campaign and reaching out directly to WV about CVD risk.^19,20^ Despite such efforts, CVD awareness has decreased among women, particularly women at higher risk for CVD (*e.g.,* WV with mental health conditions).^21,22^ Thus, exploration of additional approaches to reduce CVD risk among WV is warranted.

As an efficient and practical approach for addressing biological- and sociocultural-based CVD risk factors for WV, this article describes an approach to tailoring an existing evidence-based CVD preventive care intervention designed for Veterans without specific attention to the needs of women. Specifically, we started with the Team-supported, Electronic Health Record (EHR)-leveraged, Active Management (TEAM) program^23^ and sought to incorporate key biological risk factors for CVD unique to women and considerations of sociocultural experiences of WV and how they might increase risk or present barriers to risk reduction. We sought to start from an existing, evidence-based intervention to speed the process of tailoring and reduce the lag to impact patient care. This process for tailoring a CVD preventive care intervention for WV and adapting other CVD preventive care services may be applicable for women both within and outside of VA health care settings.

## Materials and Methods

### Study overview

This study aimed to tailor the TEAM program, accounting for biological and sociocultural-based CVD risk factors and barriers to care often experienced by WV. The interdisciplinary research team consisted of nurses, a women’s health physician, health behavior experts, and a health coach, each with experience delivering and/or studying CVD care among the Veteran population. For the purpose of this work, we adopted the following definitions. Sex refers to an individual’s anatomy, physiology, genetics, and hormones assigned at birth, while gender refers to the spectrum of an individual’s identity, roles, and relations.^24^ Use of language such as “tailoring for women” in this study is used to address both biological-sex- and sociocultural gender-specific needs within the intervention.

### The TEAM intervention

The original TEAM program is a multisite quality improvement project designed to improve CVD risk factor management among rural Veterans with an *a priori* focus on expanding access to hypertension (HTN) care and self-management support, to improve blood pressure (BP) control.^23^ Simply put, the original TEAM program offered BP management strategies without attention to those CVD risk factors that may be sensitive to biology or sociocultural experiences. TEAM is delivered virtually using a combination of existing telehealth infrastructure (*i.e.,* the VA Video Connect platform) and telephone-based outreach. Core components of TEAM include team-based care planning by a population health manager (PHM), the provision of EHR-based support to VA primary care teams, telehealth outreach, and support provision for self-management and shared decision-making. PHMs orient Veterans to their personal CVD risk profile, guide the selection of individualized HTN self-management goals using motivational interviewing techniques, identify and refer Veterans to community and/or VA-based resources (*e.g.,* weight loss programs, nutrition clinics) to support their self-management goals and monitor Veteran progress towards goal attainment. Through EHR-based communications, PHMs alert VA primary care teams to Veteran progress, including home BP trends and potential indications for changes in BP medication therapies (*e.g.,* in response to BP trends or medication intolerance). TEAM has been previously shown to lead to postprogram increases in completed medical appointments and decreases in systolic and diastolic BPs.^25^ In total, 1,110 patients enrolled in TEAM, 328 of whom (29.5%) were women.

### Tailoring TEAM for WV

Intervention tailoring consisted of several key steps, which are outlined in Table 1. First, a focused literature review was conducted to identify important sex- and gender-based differences and disparities in CVD progression and management. Second, publicly available resources for CVD risk reduction in women were reviewed to identify pre-existing patient education materials previously tailored for women. Finally, semi-structured interviews were conducted with patients who received the original TEAM intervention and TEAM providers and staff. Findings from this careful and structured process were used to inform the modification of the TEAM program. These modifications were systematically applied to existing patient and provider intervention materials. Multilevel partner engagement and input were attained from users of the original TEAM program including PHMs, primary care providers, and WV. The tailoring process occurred over 7 months from January to August, 2021, and was largely asynchronous with twice-weekly meetings for discussion and decision-making. During these meetings, provider-facing EHR templates were repeatedly reviewed by clinical team members to optimize the efficiency and effectiveness of communicating key information to best align with existing primary care provider workflow.

**Table 1.** Tailoring TEAM Patient- and Provider-Facing Materials

Source	Findings driving tailoring	Findings informing patient-facing communication changes	Findings informing provider-facing communication changes
Literature review 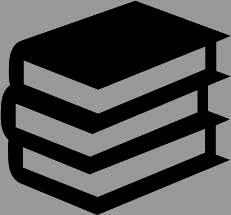	• Low awareness about CVD risk for women among patients and providers • Presence of barriers to heart healthy lifestyle due to sociocultural experiences as a woman (*e.g.,* primary caregiving responsibility as a barrier to regular exercise) • Sex-specific CVD risk factors (*e.g.,* gestational diabetes) • Clinical inertia and disparities in guideline concordant CVD care	• Messaging about CVD risk for women generally • Messaging about importance of HTN for CVD risk reduction in women • Assess women’s sex-specific risk enhancing factors for CVD (*e.g.,* pre-eclampsia)	• PHM documents women’s sex-specific risk enhancing factor in EHR note
Existing resources 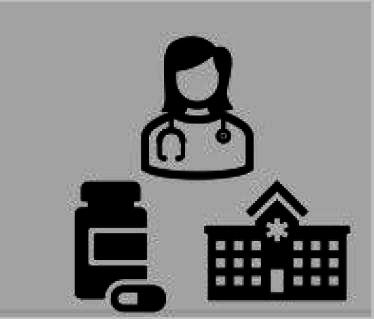	• VA delivered handouts on CVD and HTN management • Public organization materials specific to women and CVD (*e.g.,* AHA Go Red for Women) • Materials from prior HTN interventions	• Educational materials addressing women-specific (including biological sex and sociocultural gender) CVD risk and strategies to risk reduction	• Resources made available in clinic settings
Partner engagement 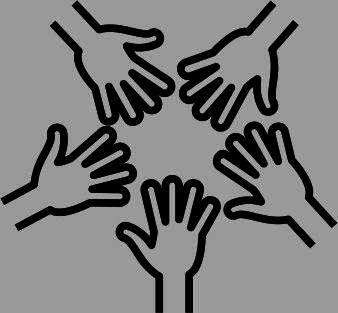	• Review of program content by women Veteran engagement panel • Review by VA primary care providers and experts in CVD risk reduction	• Tailoring of prior HTN intervention materials for women • Revision of images and messaging in TEAM materials	• Revisions to primary care team notes in EHR to document patient sex, gender, and sex-specific risk enhancing factors

AHA, American heart association; CVD, cardiovascular disease; EHR, electronic health record; HTN, hypertension; PHM, population health manager; TEAM, the Team-supported, Electronic Health Record-leveraged, Active Management; VA, Veterans Association.

#### Literature and resource review

A detailed literature review was conducted with a focus on three key topics: 1) biological (*e.g.,* pregnancy-related CVD diagnoses) and sociocultural (*e.g.,* less physical activity engagement among women) differences in CVD risk factors and experiences with CVD risk reduction efforts; 2) barriers to WV engaging in CVD risk reduction; and 3) existing evidence-based approaches to tailoring CVD prevention programs for women. Relevant literature was identified using MeSH terms for CVD and Veterans (*e.g.,* “Cardiovascular Diseases,” “Primary Prevention,” “Veterans,” and “Veteran’s Health”) combined with terms for women, sex and gender (*e.g.,* “Sex”, “Gender Identity”). Additional references from seminal articles on WV and CVD were manually searched, and recommended literature was solicited from co-investigators on this project. Biological and sociocultural factors influencing HTN inequities were considered based on their interaction with the WV population at three levels: 1) medical interventions; 2) community resources; and 3) population health approaches.

VA-developed materials, previous VA CVD risk reduction programs, the Centers for Disease Control and Prevention (CDC)/governmental websites, and AHA materials accessed through VA partnerships were reviewed, including program materials from the VA-funded CVD interventions EMPOWER QUERI^26^ and CITIES.^27^ Key materials tailored for women were identified and selected from these sources through group consensus and incorporated into the TEAM process and collection of education materials.

#### Semi-Structured interviews

WV who had enrolled in the original (un-tailored) TEAM intervention, upon completion of the program, were invited to participate in semi-structured interviews to inform the tailoring process. The interview process received Institutional Review Board approval, and all interviewees completed informed consent. Interviews were conducted *via* phone call by trained qualitative analysts (TL, AS), who did not participate in the delivery of the original TEAM program. Interview guides were based on the Chronic Care Model and program components. Participants were invited to reflect on their experience of CVD risk reduction and HTN management and their experience navigating BP self-management as a woman. Questions from the interview guide included questions such as, “In what ways do you think the TEAM Program helped you make improvements in your health/lifestyle?” and “What conversations have you had with your provider(s) about heart health risks related to uniquely female health topics, such as pregnancy?” Qualitative analysts generated notes using templated interview guides. Interviews were conducted in an ongoing manner throughout the duration of the delivery of TEAM, with the final sample size guided by the concept of information power.^28^ A similar approach was applied to interviews with TEAM program staff.

#### Modification of TEAM provider and patient focused materials

**FIG. 1.**
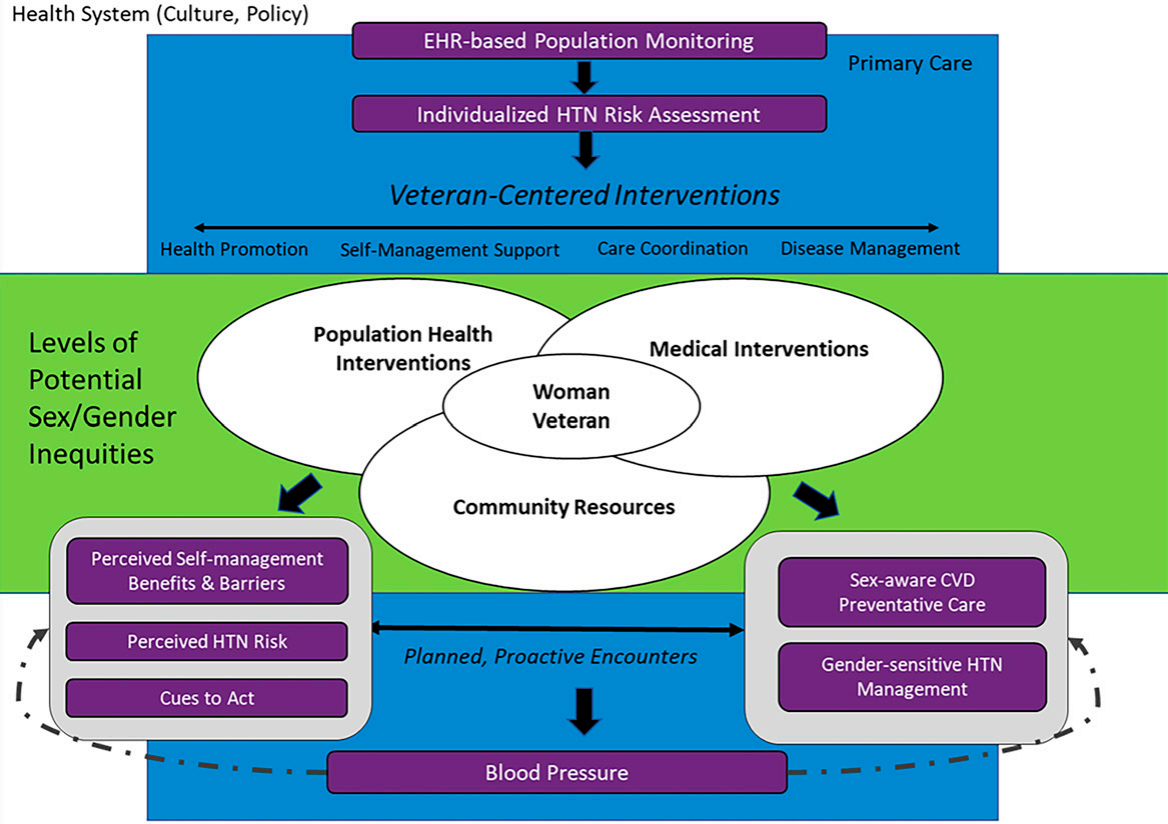
Model for adapting TEAM to address female and women-specific barriers to hypertension control. CVD, cardiovascular disease; EHR, electronic health record; HTN, hypertension.

Findings from the literature review and semi-structured interviews were used to modify patient- and provider-focused TEAM components, informed by the tailoring model (Fig. 1), the Chronic Care Model, and a population health framework.^29,30^ Patient and provider requirements were considered separately to address distinct knowledge and skill gaps.

Provider-facing TEAM components were tailored for WV with a focus on improving the identification and documentation of underrecognized biological risk factors and sociocultural experiences, as well as barriers to CVD self-management.^7^ Program materials were modified with the goal of overcoming clinical inertia in HTN management. Modifications were drafted by the clinical lead and iteratively revised by clinically trained team members until a consensus was reached. Feedback was provided by a primary care provider, a clinician with women’s health expertise, and PHMs experienced with delivering the original TEAM program.

Patient-facing TEAM components were tailored for WV with a focus on raising CVD risk awareness and enhancing education regarding risk factors related to underlying biology and sociocultural experiences that serve as risk factors and barriers to CVD risk reduction. In addition, all patient-facing materials were edited to ensure an emphasis placed on images, language, and overall messaging. Modifications were drafted by the clinical lead and subsequently circulated to the larger team and key partners for feedback and revised materials were then reviewed by the VA Women’s Improvement Network.^31^ A standing, virtual, all-women Veteran engagement group organized and supported by the VA Women’s Health Research Network was developed to provide critical input to research and programs addressing WV needs. Revised patient materials were also reviewed by TEAM PHMs for alignment with other TEAM resources and incorporation into workflow.

## Results

Overall, the process of tailoring TEAM for WV was conducted over 12 months through weekly team meetings and asynchronous review of updated materials and the outcomes of this process are described below.

### Key findings from the literature and resource review

Contemporary literature identified sociocultural experiences of WV with a potential impact on CVD risk awareness and risk reduction behaviors including inadequate social support, insufficiently perceived CVD risk, low confidence in maintaining health-related routines, stress, and difficulty balancing preventative CVD care with finances, caregiving responsibilities, and/or work.^7,18,29,30,32^ Essential sex-specific risk-enhancing factors for informing CVD risk reduction for women included the recognition of pregnancy-related conditions (*i.e.,* gestational diabetes) and menopausal status.^7,18,33^ Provider barriers to optimal CVD risk management included a lack of access to relevant patient data and referral resources, time constraints, the complexity of women’s health needs, and self-efficacy for counseling on CVD-related behavior change.^29,34^

The following themes were identified and used to inform the tailoring of TEAM for WV: 1) the need to raise women’s awareness of the importance of CVD risk profiles informed by biologically-based risk factors and recognition of sociocultural experiences common among women that may hinder CVD risk reduction; 2) the importance of emphasizing BP control for CVD risk reduction in women; 3) supporting WV providers in recognizing the influence of biology and sociocultural experience of CVD risk for women; 4) tailoring counseling and education to address sociocultural barriers to CVD risk reduction; and 5) prompting action among providers for WV to overcome clinical inertia.

### Tailoring the TEAM process to create TEAM-WV

Adjustments to the existing TEAM patient-facing materials included: 1) adding women-specific CVD risk handouts to the repertoire of resources provided to TEAM-WV participants as sourced from existing reputable organizations (*e.g.,* AHA, CDC); 2) emphasizing the relevance of CVD risk to women through directive statements regarding prevalence, the burden of disease, and highlighting examples of sex-specific CVD risk factors unique to the biology of women; 3) assessing and providing education on sex-specific CVD risk profiles for each participant (*e.g.,* pregnancy related CVD diagnoses); 4) incorporating discussion regarding the sociocultural experiences associated with women’s gender-based HTN self-management barriers into PHM call scripts (*e.g.,* competing demands due to dependent care); 5) including visual representations of women within program materials, ensuring a diversity of age, racial, ethnic, and gender identities; and 6) updating communication program materials to include gender-inclusive language, such as she/her and they/them pronouns.

**FIG. 2.**
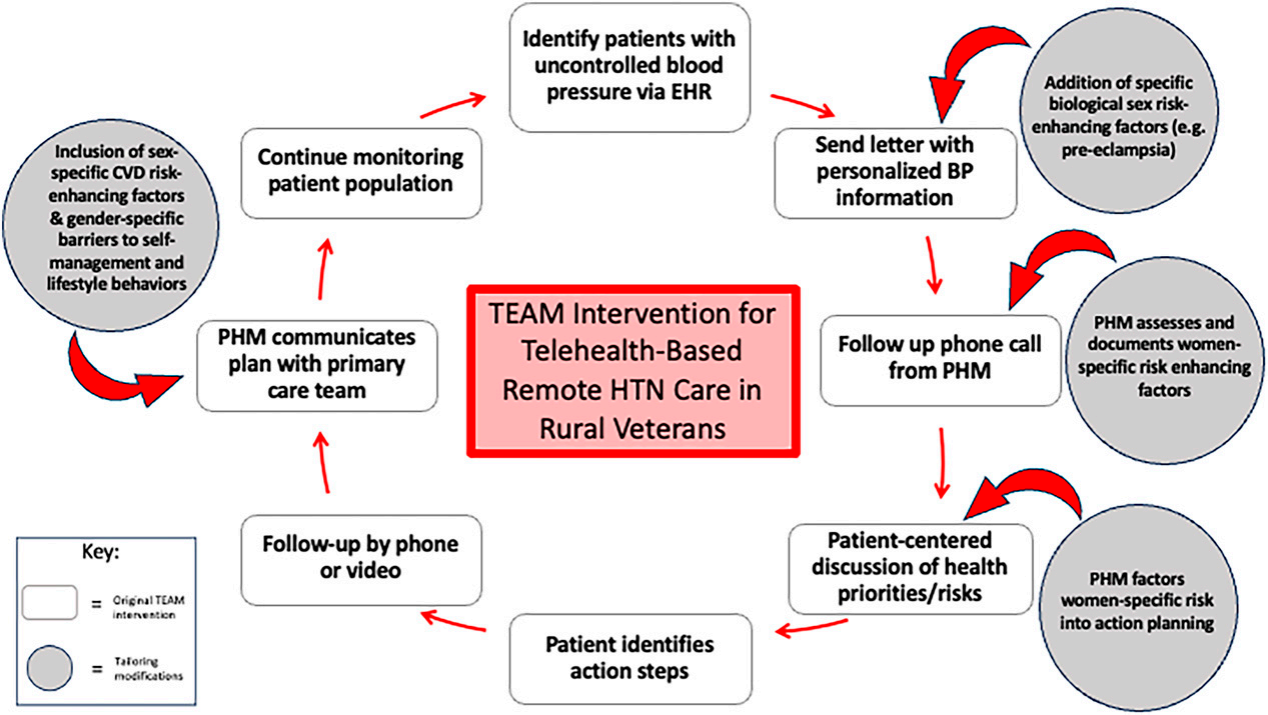
Tailoring TEAM for sex and gender-specific considerations. CDC, Centers for Disease Control and Prevention; CVD, cardiovascular disease; TEAM, the Team-supported, Electronic Health Record-leveraged, Active Management; VA, Veterans Association.

The following provider-facing communications from the PHM to VA primary care teams in the EHR were updated: 1) sex-specific CVD risk-enhancing factors based on underlying biology were incorporated into the assessment tools used by PHMs during initial contact with participants; and 2) the structured EHR communications provided to primary care teams were expanded to include documentation of sex-specific CVD risk-enhancing factors, personalized CVD risk assessment, and documentation of sociocultural influenced barriers to BP self-management. Figure 2 demonstrates the tailored adjustments made to the existing TEAM intervention. A new EHR template was created to enhance communication of WV CVD risk profiles and HTN self-management goals, with participant-identified specific, measurable, achievable, relevant, and time-bound (SMART) goals informed by sociocultural barriers to change, and actions taken by the PHM and/or recommended for primary care providers. This template included a detailed assessment of key sex-specific risk-enhancing factors for CVD in women, including gestational diabetes, cardiotoxic breast cancer treatments, and early menopause among others.^33^

## Discussion

Although individualized, patient-centered clinical practice is crucial for ensuring optimal disease and outcome management, the impact of individual’s underlying biology and/or sociocultural experiences on those conditions that occur among women and men, such as HTN, have been frequently overlooked. This article aims to describe an efficient, reproducible, and structured strategy for tailoring an evidence-based preventative care program for CVD risk to women-specific requirements. Specifically, the existing TEAM intervention was tailored to address the unique biologically-based CVD risk factors and common sociocultural experiences that impact WV diagnosed with HTN in a way that was much faster and easier than starting a new intervention from the ground up. This tailoring process may offer a time- and cost-effective approach for addressing these disparities among women with a range of sex-independent and genderless conditions.

The influence of biology is widely acknowledged across a spectrum of chronic conditions (*e.g.,* greater prevalence of migraines among women, worse outcomes following vascular procedures among women), while understanding the impact of sociocultural differences on health is growing.^35,36^ Social norms and gender roles can impact how individuals access care, navigate health care systems, and engage in preventative health behaviors (*e.g.,* diet and physical activity).^37^ Approaches to managing these disparities, however, are lacking, and literature addressing biological and sociocultural-based intervention tailoring remains limited. Previous tailoring interventions have included awareness/education campaigns and specialized clinical programs that consider the unique biological, social, and cultural factors influencing women’s health. Recently, the National Institutes of Health (NIH) Office of Research on Women’s Health emphasized significant knowledge gaps in the evidence base for preventing, diagnosing, and treating chronic diseases in women.^38^ They proposed a framework to highlight the disparity between the prevalence, presentation, and long-term effects of chronic conditions among women and men. Future research priorities will be required to align with the unique needs of women with chronic diseases to comprehensively address women’s long-term health. Heightened understanding and awareness of chronic disease is a particularly pressing need among the aging WV population, as nearly 60% of WV aged under 44 years and 75% of those aged 44–65 years have been diagnosed with at least one of the leading CVD risk factors.^39^ Furthermore, the recent launch of the White House Initiative on Women’s Health Research underscores the need for innovative solutions to health disparities experienced by women.^40^ In this study, collectively accessing medical, community, and population health resources provided a valuable opportunity to review sex- and gender-based information from a range of sources. Community resources, for example, often offer weight loss or exercise programs designed for women which can provide peer support from women with similar life experiences. In contrast, population health approaches offer strategies to identify, target, and monitor patterns of disease control at a much larger level.

While the current study addresses biological and sociocultural-tailoring for WV with HTN, many chronic conditions, such as diabetes, cancer, or arthritis, could benefit from similar intervention tailoring. Oftentimes, incorporating biological and sociocultural considerations may not require novel intervention development but rather a cost-effective modification of existing programs that closely meet the needs of women. While efficacy may need to be reestablished after tailoring, utilizing existing interventions could accelerate the process and the time to clinical implementation. Importantly, maintaining fidelity to the original evidence-based protocols while incorporating cultural content or engagement methods to tailor to the needs of women, is crucial.^41^ Systematic application to all patients, however, will be required to overcome implicit bias and poor awareness among clinical teams. Clinicians, researchers, and public health leaders should document health program tailoring approaches for meeting the needs of women to promote the dissemination of information and guide the development of this field. Additional minority groups, such as transgender and gender-diverse Veterans, may also benefit from a similar approach to tailor CVD interventions toward the needs of this population. The omission of gender-diverse or transgender individuals from this study is a limitation, however, this model offers a framework for tailored approaches that could be applied to these groups.

Multimethod evaluation of TEAM-WV is currently underway, including qualitative interviews with PHMs and WV and chronicling individual site changes to the tailored program materials. Future analysis will include a comparison of BP changes in WV pre- and post-implementation of TEAM-WV. An audit of implemented gender-tailored changes at each site and an assessment of the number of VA and community-based services Veterans are connected to through TEAM-WV will also be conducted.

## Conclusion

Tailoring of interventions for chronic conditions experienced by women and men alike is a feasible and structured approach to leverage existing evidence-based programs to address health disparities for women. The tailoring process includes drawing on extant literature, existing women-specific public health resources, and iterative input from key program partners. As health care providers strive to achieve equitable health care outcomes, interventions that address differences due to biology and sociocultural experiences must be prioritized to ensure that the diverse needs of all populations are adequately addressed. Results from this study highlight adaptation considerations for other CVD preventive care services for women within and outside of VA health care settings, as well as addressing interacting biological, social, behavioral, and health system risk factors that impact outcomes among women with chronic conditions.

## Abbreviations Used

AHA: American Heart Association
BP: Blood pressure
CDC: Centers for Disease Control
CVD: Cardiovascular Disease
EHR: Electronic Health Record
GDMT: Guideline-directed medical therapy
HTN: Hypertension
MV: Male Veterans
NIH: National Institutes of Health
PHM: Population Health Manager
TEAM: Team-supported, Electronic Health Record-leveraged, Active Management
TEAM-WV: Team-supported, Electronic Health Record-leveraged, Active Management for Women Veterans
VA: Veterans Health Administration
WV: Women Veterans
